# The story of pain in people with dementia: a rationale for digital measures

**DOI:** 10.1186/s12916-025-04057-3

**Published:** 2025-04-17

**Authors:** Monica Patrascu, Line I. Berge, Ipsit V. Vahia, Brice Marty, Wilco P. Achterberg, Heather Allore, Richard R. Fletcher, Bettina S. Husebo

**Affiliations:** 1 Centre for Elderly and Nursing Home Medicine, Department of Global Public Health and Primary Care, University of Bergen, Bergen, Norway; 2 Neuro-SysMed Center, Department of Global Public Health and Primary Care, University of Bergen, Bergen, Norway; 3 NKS Olaviken Gerontopsychiatric Hospital, Askøy, Norway; 4 Division of Geriatric Psychiatry, McLean Hospital, Belmont, MA USA; 5 Department of Psychiatry, Harvard Medical School, Boston, MA USA; 6 Department of Public Health and Primary Care, LUMC Center for Medicine for Older People (LCO), Leiden University Medical Center, Leiden, The Netherlands; 7 Yale School of Medicine and Yale School of Public Health, New Haven, CT USA; 8 Mobile Technology Group, Department of Mechanical Engineering, Massachusetts Institute of Technology (MIT), Cambridge, MA USA

**Keywords:** Pain, Pain assessment, Older adults, Dementia, Sensing technology, Digital phenotyping, Challenges

## Abstract

**Background:**

The increasingly older world population presents new aging-related challenges, especially for persons with dementia unable to express their suffering. Pain intensity and the effect of pain treatment are difficult to assess via proxy rating and both under- and overtreatment lead to neuropsychiatric symptoms, inactivity, care-dependency and reduced quality of life. In this debate piece, we provide a rationale on why valid digitalization, sensing technology, and artificial intelligence should be explored to improve the assessment of pain in people with dementia.

**Main text:**

In dementia care, traditional pain assessment relies on observing the manifestations of typical pain behavior. At the same time, pain treatment is complicated by polypharmacy, potential side effects, and a lack of around-the-clock, timely measures. But proper pain treatment requires objective and accurate measures that capture both the levels of pain and the treatment effects. Sensing systems research for personalized pain assessment is underway, with some promising results regarding associations between physiological signals and pain. Digital phenotyping, making use of everyday sensor data for monitoring health behaviors such as patterns of sleep or movement, has shown potential in clinical trials and for future continuous observation. This emerging approach requires transdisciplinary collaboration between medical and engineering sciences, with user involvement and adherence to ethical practices.

**Conclusion:**

Digital phenotyping based on physiological parameters and sensing technology may increase pain assessment objectivity in older adults with dementia. This technology must be designed with user involvement and validated; however, it opens possibilities to improve pain relief and care.

## Background

Victor is 79 years old. A few years ago, he suffered a stroke, leading to severe vascular dementia, aphasia, paralysis on the right side of the body, and neuropathic pain in his right shoulder. When he experiences pain, he is not able to communicate it but reacts with agitation and vocalizations indicative of fear. Victor is a typical patient in a Norwegian nursing home, where most of the residents have chronic complex conditions, which often include neurological diseases such as stroke or dementia.

Whether in nursing homes, hospitals or private residences, Victor’s situation is becoming more frequent worldwide. The universal reason is that people are living longer than ever before [1]. This good news comes at a price. Multimorbidity, dementia, chronic pain, and polypharmacy are increasing with advanced age and manifest with heterogeneity across various cultural groups [2–4]. The global prevalence of dementia is expected to rise from 57 million (2019) to 152 million people (2050), with Alzheimer’s disease being the most frequent etiology [5, 6]. The Long-term Care Report 2021 describes how Europe has undergone a fundamental change in the age-dependency ratio with fewer young workers needing to support a growing number older adults [7]. The existing deficit in available caregivers stands at 7 million in Europe and is estimated to triple by 2050 [8]. This deficit cannot be filled by informal caregivers alone; there is an urgent need to develop innovative approaches that address this gap while enhancing proper treatment and care [9, 10]. And as the coronavirus disease 2019 (COVID-19) pandemic has recently shown, isolation and loneliness can boost depression, pain, psychosis, and undignified death [11–13]. This means that pain is not only connected to the physical status of a person but also related to their social, spiritual, and psychological experiences.

In this debate piece, we focus on pain, which, due to high prevalence worldwide [12, 14], drives considerable costs for both society and individuals [15]. The prevalence of undiagnosed and untreated pain in nursing home patients with dementia is high, with around 43% to 80% experiencing clinically significant pain [14, 16, 17]. There are many different causes, such as pain related to the musculoskeletal system, internal organs, head, and skin. Genitourinary infections and wounds, such as pressure ulcers are regularly observed in nursing homes [18]. For this vulnerable group, chronic pain is frequently accompanied by a pain avoidance behavior, which ultimately leads to less movement and more pain [19]. Of particular interest is orofacial pain, which is related to poor oral health care, especially at the end of life [20, 21]. The Resource Use and Disease Course in dementia—Nursing Home (REDIC-NH) study recently demonstrated that the proportion of patients with ≥ 6 oral symptoms increased from 16% when perceived as dying to 20% on the day of death [20]. On the day of death, 66% experienced xerostomia, 59% dysphagia, and 50% mastication problems. Not all patients experiencing pain have chronic pain (pain duration ≥ 3 months), but those who do are more likely to have an accelerated memory decline [22]. Using the Mobilization-Observation-Behavior-Intensity-Dementia-2 (MOBID-2) Pain Scale, Pain Assessment in Advanced Dementia Scale (PAINAD), or self-report scales (depending on participants’ communication capabilities), another study found nociceptive pain as the most prominent type (70%) in nursing home patients, followed by a mix of nociceptive and neuropathic pain (25%) [17]. There is no difference in pain between dementia subtypes, but people with more severe dementia experience pain more often than those with less severe dementia (27% vs 15%) [17].

These pain details are of key importance for people with dementia because untreated pain may be a trigger for neuropsychiatric symptoms such as agitation, psychosis, depression, and sleep disturbances [23, 24]. People with dementia cannot easily articulate their suffering, as they often have speech impairment, do not remember or expect the pain; they are also frequently unable to acknowledge the impact of treatment and the potential side-effects of medication. This means that pain assessment has to rely on the observation of a caregiver, i.e., proxy rating, which uses visual or auditory cues such as vocalizations, facial expressions, or body language. During the last 40 years, more than 35 proxy-rating pain assessment instruments have been developed and tested to address this need, but they may have low validity in clinical practice and require the skills from a rater who knows the patient’s usual behavior [16].

In this debate paper, we argue for the importance of investigating non-invasive, unobtrusive, and ubiquitous methods of pain estimation for the vulnerable group of older adults with dementia as an approach to achieve more valuable pain scores and in the very end better pain management.

## Challenges of pain assessment

For older adults and especially for people with communicative and cognitive impairment, such as dementia, pain is investigated through various lenses: biological perspectives, assessment challenges, education, and management [25]. Traditionally, in clinical practice, their pain assessment requires a proxy-rater, usually the primary nurse, which increases the risk for over or underestimation. Even for younger adults, chronic pain is difficult to self-quantify because of its permanence and subjectivity of experience. When combined with other conditions and cognitive impairment, pain manifests in ways often impossible to disambiguate or measure accurately.

One might ask, “But why does pain assessment matter?” pointing at the plethora of available analgesics. “If the underlying pain-causing condition is known, would it not be expected that pain treatment follows?”.

One of the main pain treatment challenges for older adults is the overprescribing and overuse of medication, especially in nursing homes. About 65% of nursing home residents receive some form of analgesic regularly but often without properly testing pain intensity before or after treatment [14]. This is a particular concern when it comes to opioids [26]. For adults without cognitive impairment, although they might be able to express their pain, there is still not enough comprehensive knowledge related to the interactions between different drug profiles, especially for combinations of three or more medications [27–29]. For people with dementia, the side-effects of opioids in combination with other centrally active drugs can be difficult to detect and negatively impact the patients’ activities and function. For instance, a placebo-controlled study by Erdal et al. demonstrated considerable negative side-effects of buprenorphine when combined with antidepressants [30], while failing to find a positive effect of individual treatment on pain [31]. Non-pharmacological pain therapy approaches are difficult to evaluate for efficacy [32] or often fail to have an impact on pain, as was recently shown for a music-based intervention [33].

Circumstances make proper pain assessment extremely challenging because pain cannot be easily recognized or quantified by an external observer and the typical behavior for pain may be identical to the typical behavior related to dementia. It is almost impossible to see, with the naked eye, whether another person experiences pain or not. This is especially the case for people with chronic pain because of habituation and masking [19]. This means that proxy rating has low validity and reliability, while clinical intervention trials are impaired by the placebo effect, attention bias, the Dunning-Kruger effect (overestimating one’s own knowledge), and the Hawthorn effect (adjusting behavior when being observed) [34].

For Victor and others like him, their care needs might differ in various aspects (e.g., indications for treatment, comorbid conditions, supervision, and physical and social activities), but not in complexity. People with dementia require a carefully curated and coordinated list of interventions, complicated by the fact that their effects are not easily quantifiable. And while we can objectively measure variables such as blood pressure, movement, or skin properties, the objective measure of pain remains elusive due to its hyper-personal nature.

## The technology of the current era

“Pain is complex and not fully understood” is the first general statement in many articles dealing with new methods of pain assessment via sensors [35, 36]. In the engineering sciences literature, this is an indication of an issue worth investigating. But while the clinical world is in dire need of new assessments, the technical universe provides solutions by the milligram—not for a lack of trying, but because of the complexities of human bodies, pathologies, multimorbidity, and care pathways.

In medicine, a traditional pain assessment scale produces data in form of scores, usually a number, which express the intensity of pain; e.g., Numeric Rating Scale (NRS), range 0–10 with 0 for no pain and 10 maximal pain. It is straightforward, easy to understand. By contrast, in data science and sensing, data is a signal expressed in volts, amperes, or other units, which is provided by a sensor These signals gain meaning and become information only after processing and, by enhancing them with the contextual interdependencies of their clinical background, we obtain knowledge [37].

For pain estimation, the design of new methods requires transdisciplinary collaboration with at least three steps: acquiring a raw sensor electrical signal (data), processing this signal to derive meaning (information), e.g., movement or behavior, which is then associated with pain (knowledge). The translation of electrical signals into pain is long, complex, and highly dependent on the observable manifestations of pain. While the exact evaluation of the type, duration, and intensity of pain in people with advanced dementia is not guaranteed, the clinical experiences (knowledge) describe typical pain behavior, for instance agitation, aggression, defensive movements, vocalization or typical facial expressions of pain [38].

According to [39], some of the most investigated data sources in pain estimation range from wearable-driven physiological signals to functional near infrared spectroscopy. While their pre-processing is often based on signal processing techniques, the algorithms for pain estimation are machine learning to construct a classifier for the presence of absence of pain on categorical levels, such as low, medium, or high. Table 1 gives an account of the most common unobtrusive wearable and environmental types and their sensors in this field [39–42].

**Table 1:** Common wearable and environmental sensors used in pain estimation

Device	Sensor	Data or estimations
Wristband, smartwatch, smart ring, socks, insoles	Photoplethysmogram (PPG) Electrocardiogram (EEG)	Heart rate, heart rate variability, blood volume pulse, respiration
	Accelerometer, gyroscope, magnetometer	Movement, activity levels, number of steps
	Electrodermal activity sensor	Electrodermal activity, stress response
	Temperature sensor	Skin temperature
	Sensor combinations	Sleep patterns
Smartphone	Battery monitor, screen time monitor	Behavior patterns
	Accelerometer, gyroscope, magnetometer	Number of steps
Belts (thoracic, waist, limbs)	Photoplethysmogram (PPG) Electrocardiogram (EEG)	Heart rate, heart rate variability, blood volume pulse, respiration
	Accelerometer, gyroscope, magnetometer	Movement, activity levels, number of steps
	Electromyography (EMG)	Muscle activity
Environmental (wall, nightstand, ceiling, furniture)	Radar	Movement, breathing, sleep patterns
	Radio	Movement, sleep patterns, pose
	Proximity sensors	Posture, movement

Current research includes trials and case studies on how everyday data can shed light on behavioral symptoms triggered by untreated pain. Actigraphy, for instance, has been applied to digital monitoring of physical movement in dementia research [30, 43, 44]. In a recent review, Werner et al. summarize automatic pain detection approaches [36]. A considerable amount of effort has been put into video-based approaches, which might be suitable for persons with full facial mobility [45] (thus excluding people with e.g., Parkinson’s disease). However, beyond the ethical and privacy considerations of filming persons who might not be able to consent (e.g., with advanced dementia), the field of facial recognition itself is still in its infancy [46]. Nevertheless, contact and contactless methods based on, for instance, electrodermal activity or heart rate variability are promising [35, 47, 48], but more investigation is needed for older adults, whose physiological responses and skin properties change as they age.

When it comes to the other manifestations of pain, symptoms such as agitation, aggression, or apathy can complement the insight a directly measurable bio-signal offers into the story of pain [49, 50]. Ambient and environmental no-contact sensors can provide behavioral information related to pain [51] (Table 1). Inferring pain information from physiological signals remains challenging, as some dementia-related behaviors are indistinguishable from those triggered by pain, and the sensing degrees of freedom do now allow for perfect disambiguation of their correlates. This is where knowledge (as necessary for medical applications) must be the result of co-creation, of combined efforts between clinical experts, engineers, and user involvement. Because of their complexity, these designs usually involve artificial intelligence (AI) methods, which come with their own pitfalls and most often focus on diagnosis and prevention of various diseases, including dementia, and less on symptom tracking [52].

The potential, however, is there, as we move away from data-driven to knowledge-driven artificial intelligence. As Andrew Ng says in his interview, Unbiggen AI, for IEEE Spectrum in 2022, “In many industries where giant data sets simply don’t exist, I think the focus has to shift from big data to good data” [53]. And for medicine, good, multi-modal/multivariable data comes very close to knowledge that is only useful in the hands of skilled, human caregivers.

## The future of pain assessment in people with dementia

We can imagine a future in which Victor’s pain is recognized and addressed more easily. Victor wears a watch on his wrist, showing him the time of day; underneath that display, his pulse, respiration and electrodermal activity are logged and processed, alongside his movement. As his caregivers tend to him, they observe his behavior and reactions, evaluating the way he moves, facial expressions, vocalizations, etc. Adding to this snapshot assessment and for the periods when they cannot be near Victor, they are also able to access indicators that describe differences in activity patterns throughout the day or identify periods of increased stress response. Together with their own observations, they can better monitor when Victor’s pain is increasing or how he responds to treatment. The monitoring is unobtrusive, while the digital measures inform clinical decisions, still in the capable hands of healthcare providers. This use-case scenario might not be far in the future, but there are still steps to be taken in the meantime.

Digital phenotyping was introduced as an information-driven method of phenotyping, which is the description of observable characteristics of an organism. Although the term digital phenotyping is commonly associated with smart phone data, any data that is ubiquitously and continuously collected from wearable sensors (e.g., smart watch) or devices in our environment (e.g., motion sensor) can be processed so that meaningful digital biomarkers provide insight into various conditions [54, 55]. With this real-time, individualized analysis, health care can benefit from increased temporal resolution and dynamic prediction of symptom trajectories, both in and outside of clinical settings. In the internet-of-things (IoT) era, it can be expected that personal wearables will join the inter-device communication network to better shape the understanding of our mental, physical, and social landscapes.

Right now, digital phenotyping resides at the intersection between wearable and environmental sensing, clinical knowledge, signal processing, and artificial intelligence. Its emergence in the medical research field has been met with both skepticism [56] and enthusiasm [57], but herein lies the future. If sensing can give machines a voice, why not allow it to help those who have lost their own? Wearable or ambient sensors in the home can passively monitor and detect a person’s movement-related behaviors that are indicative of pain. Borne silently or loudly, pain is an essential symptom across the entirety of health care, and for those unable to express it, digital phenotyping can provide new assessment tools [58].

With public and patient involvement, we must consider what vulnerable older people with dementia need and want from technology and their living environments. These older adults have increased risks of loneliness and institutionalization, while their unmet needs are of both medical (quality of care) and social (quality of life) nature. Pain should not contribute to the problem. Furthermore, future designs must account for what the primary care system needs in terms of skills, competences, infrastructure, investments, and the unexpected bottlenecks of emerging technologies. For clinicians and engineers alike, the users must be both patients and caregivers (formal or informal), and so user-centric co-creative development is essential.

## The other side of the technology medal

In addition to technical innovation, it is also important for research to identify critical factors in the validation of new welfare technologies for pain evaluation, such as artificial intelligence. We must also consider the role research should take in collaboration with users and industry, e.g., pharmaceutical industry. The success of machine intelligence in other fields has found fertile ground in medical big data; however, this has opened the doors to the dangers of under-validated tech, with long-term consequences. As it is, industry has its own interests, and we point out that developers must bear the responsibility to ensure validation of their products, transparency, data access, privacy protection, and misuse prevention. The market-driven, fast pace of tech progress means that the too-many short-lived products (by validation they are already obsolete) raise issues of sustainability and mistrust from healthcare stakeholders. Wearables themselves require validation as there is no standardized regulation for the quality and other properties of the data collected by these devices.

Investigations, design, and implementation of digital measures for symptom assessment do not stop at pain. These advances have ethical implications across healthcare. A smartwatch, for instance, is a multimodal sensing device, and as such it can be useful in tracking and combining other symptoms or making predictions about changes in health status. In this context, we must define the boundary between monitoring and surveillance, from the perspective of personal privacy, dignity, and safety, on the backdrop of new legislation being designed to address the new ethical issues raised by artificial intelligence [59]. This investigation is critical to the future of technological solutions.

For good reason, “too much technology” is described as dangerous [60]. In 2014, Stephen Hawking warned that the development of artificial intelligence could accelerate the decimation of jobs in traditional manufacturing [61]. But just 10 years later, European policymakers cheer the interest in digitalization as preferred solution for care gaps [62–65]. Currently, we are witnessing an engaged, polarised discussion on the relationship between technology, healthcare, and different user groups, e.g., [60, 66, 67].

Back to Victor and his care needs—We have to allow critical questions to be asked, on whether technology and data science, particularly artificial intelligence, are really the solution to issues such as pain assessment and the efficacy measure of pain treatment. What is lost and what is gained for users, healthcare systems, research, and industry? Which technologies can really address gaps and how do they need to be supported? We have not set out to answer these here, but we should bear them in mind going forward, as they will dictate the landscape for future tech development in medicine.

## Conclusions

In this debate paper, we provide a critical argument on why valid digitalization, sensing technology, and artificial intelligence should be explored to improve the assessment of pain in older adults with communicative and cognitive impairment, such as dementia. Direct measures that complement insufficient traditional proxy rating are possible via the digital phenotyping route, but these new technologies must be designed with user involvement, including clinicians and other caregivers, and must take into the account the specificities of this vulnerable group. The implications for older adults with chronic pain are major, hinting at improved care, timely treatment revisions, and better quality of life.

## Data Availability

No datasets were generated or analysed during the current study.

## Abbreviations

AI: Artificial intelligence
COVID- 19: Coronavirus disease 2019
EEG: Electrocardiogram
EMG: Electromyography
IoT: Internet-of-things
MOBID- 2: Mobilization-Observation-Behavior-Intensity-Dementia-2
NRS: Numeric Rating Scale
PAINAD: Pain Assessment in Advanced Dementia Scale
PPG: Photoplethysmogram
REDIC-NH: Resource Use and Disease Course in dementia—Nursing Home
